# Influences of eye gaze cues on memory and its mechanisms: The function and evolution of social attention

**DOI:** 10.3389/fpsyg.2022.1036530

**Published:** 2022-10-14

**Authors:** Xiyang Yin

**Affiliations:** Department of Student Affairs, Mental Health Education and Consultation Center, Guangzhou Huashang Vocational College, Guangzhou, Guangdong, China

**Keywords:** gazing cue, social attention, visual working memory, long term memory, theory of mind

## Abstract

During evolution, humans have formed a priority perceptual preference for others’ gazes. The gaze direction of others is called the gaze cue, conveying environmental information, a critical non-verbal communication in early humans. Recently, empirical evidence has indicated that gaze cues can affect high-level cognitive processes, such as memory. Unlike non-social cues (e.g., arrows), gaze cues elicit special social attention. Research determining the underlying mechanisms suggests that social intention influences observers’ visual attention and influences their memory. This article provides a brief review of the current state of research on the relationship between gaze cues and memory. Future studies should focus on multiple gaze cues, the social nature of gaze cues, and clinical research.

## Perceptual priority of eye gazes

During social interaction, gaze following is a phenomenon in which individuals unconsciously shift their social attention to follow the gazers’ orientation (Mansfield et al., 2003; Dalmaso et al., 2020). Hence, the orientation of others provides a gaze cue. This evolutionary cue triggers social cognition, which allows individuals to acquire surrounding information (Sun et al., 2017). Evidence shows that eye gaze can orientate others’ attention and obtain priority visual processing (Hoffman and Haxby, 2000). Gaze-following is one of the first and most essential steps in engaging in social communication (Csibra and Gergely, 2009). By 3 months, infants can follow an adult gaze orientation without the gazers’ head orientation (Hood et al., 1998). Further, at 3 years of age, children can fairly accurately evaluate what individuals are looking at (Doherty, 2006). In adulthood, this evolutionary cue triggers socio-cognitive processes, which allows individuals to engage in more social cooperation by inferring others’ behavioral intentions and mental states (Sun et al., 2017). Although adults can use other rich social skills to complete social interactions, relying on gazing cues to guide and conduct social behavior is still the core content across the human lifespan. Recently, researchers found that gaze cues as a special selective attention (Wang et al., 2019), can closely interact with working memory (Nie et al., 2018; Gregory and Kessler, 2022; Lee and Pitt, 2022). Research evidences demonstrated that gaze cues modulate the observers’ attentional distribution, and modified their memory capacity, to enhance item discrimination. So far, to the best of our knowledge, no available review in this field of studies has been conducted. Gaze cues play an important role in non-verbal communication during human social interactions (Vaish et al., 2017). The memory enhancement of gaze cues might have an evolutionary implication that is associated with the social tunning effect (Shteynberg, 2010).

### Evolutionary advantages of eye gazes

The eye is a particular human sense that has evolved to enhance survival and allow humans to detect dangerous information in the environment (Yorzinski et al., 2014). In the process of evolution, human beings have formed a greater sensitivity and perceptual preference for others’ eye gaze, utilizing gaze direction to enhance cognitive processing. Evolutionary psychologists hold that, similar to biological structures, some specific human beings’ motivations are also due to the evolution of natural selection. This could be dated back to the survival pressure confronted by primitive humans in the early human stage (Cosmides et al., 1992). Human eyes have evolved a wide white sclera encompassing the darker iris and pupil. These morphological features are vital for humans to form face and gaze perceptions (Kano et al., 2022). Some psychological researchers hold that the human brain has evolved a psychological mechanism to detect eyes in the surroundings with a priority perceptual preference for others’ gaze direction (Driver et al., 1999; Frischen et al., 2007; Wu et al., 2014; McKay et al., 2021), which reflects a social-biological response to gaze cues (Emery, 2000). Interestingly, individuals’ attention can also be guided by the eye-gaze cues of cross-species (e.g., dog gaze cues), suggesting the evolutionary implications of gaze cues (Corneille et al., 2009).

### Gaze cueing effect

Friesen and Kingstone (1998) first used the gazing cues in the traditional Posner cueing task (e.g., arrows). A face on the central screen gazed left, right, or straight ahead, and a probe item was subsequently displayed either gazed at (valid trail), gazed away from direction (invalid trail), or kept the face ahead (neutral trail), which is also called joint attention (Nummenmaa and Calder, 2009). The observers were informed that the gaze direction cannot predict the target location. However, the participants still automatically followed the gaze orientation. The probe detection was more rapid and had less error in the cued than in the invalid and natural trails. Moreover, several literatures suggest that individuals could automatically process another people’s eye gaze without consuming psychological resources (Visser and Roberts, 2018).

## Social cues and memory

The social cues could trigger socio-cognitive processes, and enhance the engagement with the external environment (Sun et al., 2017). Before the paradigm of gaze cueing on memory, researchers established the joint attention paradigm to examine how social cues impact on memory. For instance, Richardson et al. (2012) elicited joint attention through a “looking together” task. In their experiment, researchers manipulated participants’ beliefs that another unseen participant whether look the same picture or not. They found that when the participants believed that the unseen participant was looking at the same picture (joint attention), memory performances were better than when the participants believed that the unseen participant was not looking at the same picture. This research indicates that imagined social cues (such as an unseen participant) can improve participants’ memory performance. Using a similar task, Shteynberg (2010) found that individuals’ memory performances were better when the stimuli were experienced by the similar person than dissimilar person, even when intragroup verbal communication is absent. It should be noted that these kinds of social cues did not explore joint attention in a natural and ecological way.

## Gaze cueing effect on memory

### Visual working memory

Visual working memory (vWM) offers a significant contribution to the formation of consistent and stable visual representations of the outside world. It allows individuals to adapt to a constantly changing environment, which stores and manipulates brief visual information for a few seconds in a temporarily available state for ongoing cognitive tasks (Baddeley, 2012). Increasing evidence indicates that visual working memory capacity is not unchangeable but can be modified or shaped during the encoding or maintenance interval.

Gaze cues can guide spatial attention, accelerate the detection of stimulus targets, and enhance the discrimination of probe items. Researchers (Gregory and Jackson, 2017) modified the classical gaze cueing paradigm and vWM task to investigate the impact of gaze cueing effects on vWM. In their study, a centrally presented face gazed at the left or right initially, and a memory array (four color squares) subsequently appeared on either the cued (gazed at the targets) or uncued (gazed away from the targets) side during the encoding interval. Participants were asked to recall whether the probe item was present during the encoding interval. Memory performance was measured using the *d*’ values originating from the signal detection theoretical sensitivity measurement. It was found that participants’ vWM performance was more accurate in the cued than in the uncued condition. However, the arrow cue did not repeat the task results. The neurocognitive evidence also found the critical difference between gaze cues and arrow cues when they were present in the vWM task. Researchers employed electroencephalography (alpha: attention; theta: effort) to reveal the cognitive neurological differences between gazing and symbolic cues in vWM (Gregory S. E. A. et al., 2021). Taken together, the behavioral and neurocognitive studies both suggest that gaze and arrow might separately operate in the different cortical networks in the vWM task. Additionally, researchers have constantly extended this series of investigations to determine how the social context modulates the gaze cueing effects on vWM. For example, when western observers looked at Japanese gazes, happy gazing cues facilitated in vWM were replicated. Contrastingly, Japanese observers looking at Caucasian gazes showed no effect of gaze cues on vWM for happy faces (Gregory et al., 2020).

The intrinsic value of gaze cues in social cognition is their remarkable flexibility (Sun et al., 2017). When the gaze cues were displayed before the presentation of memory items, it can help individuals shape external representations in the valid cued direction. Moreover, the gaze cues also modulate already-stored internal representations. Nie et al. (2018) conducted a retro-gaze cue in the vWM paradigm to determine whether observers could remember the memory array after a non-predictive gaze cue was displayed during the maintenance interval. In this task, gaze cues were presented in the maintenance interval of working memory. A gaze cue at the right or left side was preceded by a memory array display for 250 ms. Participants were asked to discriminate whether the probe item was the same as the previous item. This revealed a memory advantage for the valid gaze-cued vs. invalid condition. Further study found that motion (non-social cues) and reverse gaze cues did not change vWM similar to the gaze cue. These findings indicate that social cues (e.g., gaze cues) enhance vWM performance both in the encoding and maintenance intervals, suggesting that gaze cues play an essential role in allocating observers’ attention (Gregory and Jackson, 2017; Nie et al., 2018).

### Long-term memory

As described above, the memory effect of joint attention, caused by social cues, should be discussed in a natural and ecological way. In order to fill the gap, research on the gaze cueing effect was conducted in long-term memory. Kim and Mundy (2012) explored how gaze cues affect long-term memory (such as images). The results showed a better memory performance for images associated with the joint attention condition. However, it should be noted that in this experiment, the observers were informed to select the gazing cue, so this did not determine the automatic processing characteristics of gaze cueing effects to use (or ignore) gazing cues. Subsequently, researchers tried to combine the classical gaze cueing task without informative cues in the long-term memory paradigm. This is also the first study to extant the gaze following in long-term memory task with non-predictive cues. Dodd et al. (2012) showed target words displayed on a screen to the left or right of a central face cue. Observers were notified that the cue did not predict where the word would be present. After all target words were presented, observers were asked to write down the target words as much as possible. The results suggest that more words were recalled in the valid than in the invalid gazing cue trials. Additionally, if memory item duration lasts for 1,000 ms, the gaze cueing effect on long-term memory disappears. Remarkably, when the researchers replaced gaze cues with arrow cues, the arrow cues could not repeat the experimental effect on word recall in a manner comparable to gaze cues. These results indicate that gaze direction could serve more than simply guiding social orientation to improve individuals’ perception; it can enhance long-term memory psychologically (Dodd et al., 2012).

### Mechanism of gaze cueing effects on memory

Faced with limited visual attention and memory resources, individuals must select relevant stimuli from the visual scene for mentally prioritized processing (Shteynberg, 2010). This cognitive process depends on the interaction between selective visual attention and memory systems. Notably, gazing cues serve not only to increase the perception process but also raise the target’s social value upon a gazing orientation. Gaze cues can increase the affective value of objects through top-down modulation (Nummenmaa and Calder, 2009), while arrow cues fail to duplicate the effect as gaze cues do (Bayliss et al., 2010), implying the social nature of gaze cues (Bayliss and Tipper, 2006). Some researchers have argued that individuals are susceptible to objects others gaze at. Individuals tend to remember the item that is important for their goals (Altmann and Trafton, 2002; Montagrin et al., 2013). For example, although older adults show a decline in the gaze cueing effect, they still utilized gaze cues to facilitate memory encoding (Gregory and Kessler, 2022). In other words, older adults would like to follow the gaze cues to serve a goal-directed process rather than the uninformative cues.

Previous studies have employed physical or non-social cues (such as arrow cues) and found that these cues can also enhance participants’ memory performance. However, the eye-gaze server does not merely play the role of directional information. Recently, researchers examined the mental state account of the gaze cueing effect. They manipulated observers’ mental states in the paradigm of gazing at cues on memory. A closed bar blocked the gazer’s view of the memory items, and an open bar allowed the gazer’s view of the memory items (Gregory and Jackson, 2019). They found that gaze cueing effects on memory were absent in the closed bar condition but present in the open bar condition. These findings proposed the mental state account of gazing cues in memory, whereby the attentional focus of another enhances memory through high-level engagement with the other’s perspective.

Why would humans mentally tune their memories with the gazer? Evolutionary psychologists have argued that survival depends on successful social activities such as food gathering, hunting, shelter maintenance, and enemy exclusion (Wilson and Wilson, 2007). Primates are under constant pressure to develop shared psychological representations of their surroundings, allowing them to rapidly detect dangerous animals (Yorzinski et al., 2014). The more memories overlap with others, the more survival chances humans have (Tomasello et al., 2005), which is termed “the social tunning effect” (Shteynberg, 2010). The mental adaptations of memory enhancement of gaze cues would improve both personal and social fitness (Sober and Wilson, 1998).

## Conclusion and further research

In conclusion, eye-gaze cues can enhance the observers’ memory. In the process of human evolution, ancestors have evolved a preference for eye gaze to communicate with others. Humans have learned to use gazing cues to detect environmental information and improve opportunities for survival. The engagement of gazing cues is of fundamental significance in social cognition, which has evolved the automatic processing of human beings. However, non-social attention (e.g., arrow cues) cannot cause similar experimental effects as the gazing cue, indicating that gazing cues prioritize memory processing. The possible psychological mechanism behind this experimental effect could be accounted for by the mental state. Currently, studies on the gaze cueing effect have mainly focused on the behavioral level. For example, if researchers used the transcranial electric stimulation (tES) techniques (Živanović et al., 2022) to establish a relationship between the brain region of processing intention information and arrow cues in vWM task, the arrow cues would enhance individuals vWM performance as a manner of gaze cues did? In future studies, researchers should employ cognitive neuroscience techniques (e.g., neuroimaging and brain stimulation) to explore the critical difference between gaze and arrow cues (Gregory and Kessler, 2022), which will help us understand the social nature of gaze cues. Additionally, an interesting direction for the following research is to determine the gaze cues in more real-world surroundings. Gregory S. E. et al. (2021) have shown that engaging the virtual reality avatar or live person (Dravida et al., 2020) allows researchers a well-controlled experiment when providing observers to interact with gaze cue in an ecological experimental setting. In addition, the current research that uses single gazing cues as an example of social interaction patterns may fail to represent the complicated realities of group-level gaze dynamics (Sun et al., 2017, 2021). Therefore, we must reconsider how individuals select gaze following to affect subsequent cognition in a more complex setting (Capozzi et al., 2018; Sun et al., 2020).

## Author contributions

The author confirms being the sole contributor of this work and has approved it for publication.
